# B cells from anti-thyroid antibody positive, infertile women show hyper-reactivity to BCR stimulation

**DOI:** 10.3389/fimmu.2022.1039166

**Published:** 2022-10-25

**Authors:** Timea Serény-Litvai, Anna Bajnok, Viktoria Temesfoi, Jasper Nörenberg, Greta Pham-Dobor, Ambrus Kaposi, Akos Varnagy, Kalman Kovacs, Sandor Pentek, Tamas Koszegi, Emese Mezosi, Timea Berki

**Affiliations:** ^1^ Szentágothai Research Centre, University of Pécs, Pécs, Hungary; ^2^ Hungarian National Laboratory on Reproduction, University of Pécs, Pécs, Hungary; ^3^ Department of Immunology and Biotechnology, University of Pécs Medical School, Pécs, Hungary; ^4^ Lab-on-a-Chip Research Group, Szentágothai Research Center, University of Pécs, Pécs, Hungary; ^5^ Department of Laboratory Medicine, University of Pécs Medical School, Pécs, Hungary; ^6^ Department of Medical Microbiology and Immunology, University of Pécs Medical School, Pécs, Hungary; ^7^ First Department of Internal Medicine, University of Pécs Medical School, Pécs, Hungary; ^8^ Department of Programming Languages and Compilers, Faculty of Informatics, Eötvös Loránd University, Budapest, Hungary; ^9^ Department of Obstetrics and Gynecology, University of Pécs Medical School, Pécs, Hungary

**Keywords:** B cell receptor-hyperresponsivity, anti-thyroid antibodies, infertility, B lymphocytes, Hashimoto’s autoimmune thyroiditis, levothyroxine

## Abstract

Anti-thyroid antibody (ATA) positivity affects 1 out of 9 women in childbearing age and presents a significant risk for infertility. Emerging evidence indicates that alterations in the B cell receptor induced calcium (Ca^2+^) signaling could be key in the development of autoimmunity. We aimed to investigate the Ca^2+^ flux response of B lymphocyte subsets to BCR stimulation in Hashimoto’s thyroiditis and related infertility. We collected peripheral blood samples from ATA+, infertile, euthyroid patients (HIE), hypothyroid, ATA+ patients before (H1) and after levothyroxine treatment (H2), and age-matched healthy controls (HC). All B cell subsets of ATA+, infertile, euthyroid patients showed elevated basal Ca^2+^ level and hyper-responsivity to BCR ligation compared to the other groups, which could reflect altered systemic immune function. The Ca^2+^ flux of hypothyroid patients was similar to healthy controls. The levothyroxine-treated patients had decreased prevalence of CD25^+^ B cells and lower basal Ca^2+^ level compared to pre-treatment. Our results support the role of altered Ca^2+^ flux of B cells in the early phase of thyroid autoimmunity and infertility.

## Graphical Abstract

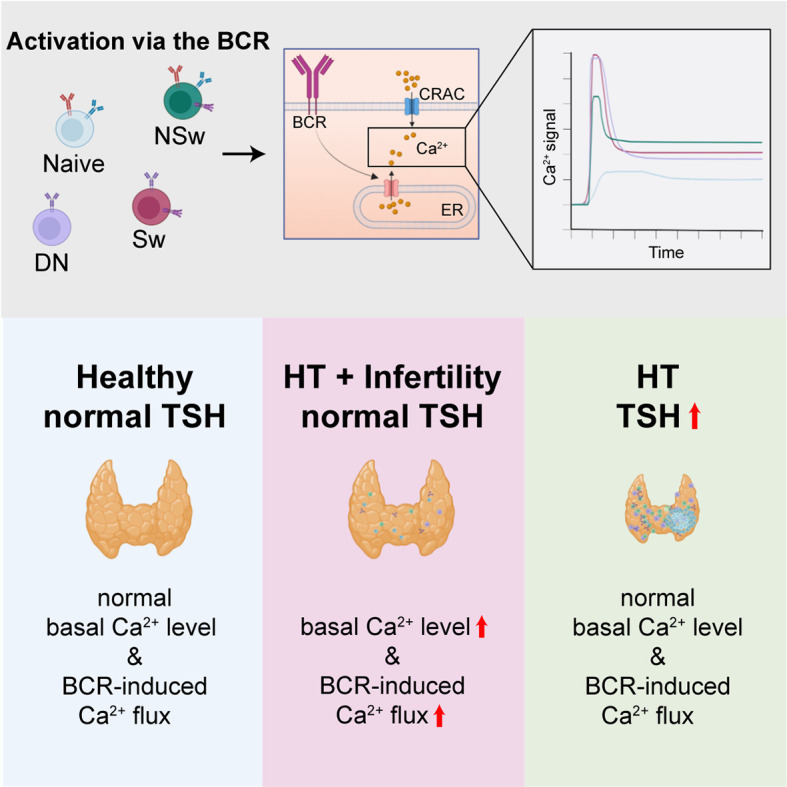

## Introduction

Hashimoto’s thyroiditis (HT) is a common organ-specific autoimmune disorder in women of childbearing age. It is characterized by the presence of anti-thyroid antibodies (ATAs), such as anti-thyroid peroxidase (anti-TPO) and anti-thyroglobulin (anti-Tg), and the infiltration of the thyroid gland by mononuclear cells (1). The complex interplay of cellular and humoral factors results in a chronic inflammatory process over several years. At a certain level of functional thyroid tissue loss hypothyroidism develops, indicated by the rising of thyroid-stimulating hormone (TSH) (1). Although the incidence of ATA positivity is between 10-14.5% in women between 20 and 40 years of age (2), the development of hypothyroidism is not obligatory, in fact the annual risk is only 2.1% (3). Therefore, despite the growing prevalence (4), the majority of ATA+ cases remain undiagnosed due to the lack of clinical signs.

This silent disease could be considered clinically irrelevant; however, ATA positivity presents a significant risk for infertility and pregnancy loss, the odds ratio (OR) of miscarriage is 3.9 even without thyroid dysfunction (5). The presence of ATAs may also have a negative impact on the success rate of *in vitro* fertilization (IVF) (6). Additionally, the immune tolerance during pregnancy also appears to be disturbed, indicated by the higher rate of preterm birth (OR 2,07 (5),) and pregnancy complications (7). The beneficial effect of levothyroxine replacement therapy during IVF and pregnancy in ATA positive, euthyroid women had earlier been shown in randomized trials, leading to great expectations (8, 9). However, a recent multi-centric, randomized, double-blind, placebo-controlled trial (TABLET) showed no positive effect of levothyroxine compared to placebo on the live-birth rate in euthyroid women with anti-TPO antibody positivity (10).

To explain these adverse outcomes regarding fertility and pregnancy in Hashimoto’s thyroiditis, two popular hypotheses have been proposed (11). First, that HT is accompanied by generalized immune dysfunction that affects the reproductive organs and impairs the maternal-fetal tolerance (12). The direct detrimental effect of ATAs has also been suggested, as they can be detected in the ovarian follicular fluid of infertile women and may lead to lower rates of oocyte fertilization (13, 14). The causal relation was demonstrated in mice immunized against human Tg that had increased fetal absorption rates and decreased weights of the fetuses and the placenta (15). Second, as elevated TSH is an independent risk factor for adverse pregnancy outcomes (16–18), that the adaptation of the inflamed thyroid gland could be inadequate to the increased demand of pregnancy (19, 20) leading to ‘relative’ thyroid dysfunction (11, 21–23). However, as levothyroxine replacement was inefficient in the TABLET trial, our attention is increasingly drawn towards the understanding of the underlying immunological alterations.

In contrast to Graves’ disease, which is widely recognized as an autoantibody-mediated disorder, Hashimoto’s thyroiditis is generally considered to be a T cell-mediated disease (1). Although the role of B cells as the source of autoantibodies in HT is clear, we have little knowledge about their contribution to the pathogenesis and their role in infertility. There is some indirect evidence of the systemic dysfunction of B cells in ATA positive women, as the prevalence of anti-ovarian and non-organ-specific autoantibodies (NOSAs) can be increased (24–26). Nevertheless, according to the classical hypothesis, auto-reactive B lymphocytes are bystanders in the inflammatory process, activated by the release of thyroid antigens in the appropriate cytokine milieu and in the presence of T helper (T_H_) lymphocytes. It is postulated, that the break in self-tolerance occurs at the level of T lymphocytes and that these antigen-specific, autoreactive T cells in turn support the activation of autoreactive B cells (27, 28). However, to date little is known about the steps leading up to the appearance of activated auto-reactive T cells, specifically because T cells require antigen presentation and specific receptor co-signaling.

The question is whether the appearance of auto-reactive, high-affinity autoantibody-producing B cells could be the first step in the pathogenesis of HT, preceding any damage to the thyroid itself. Recent advances in the field of autoimmunity have revealed that dysregulated B cell signaling *via* the B cell receptor (BCR) could be the key step and the primary driver of the loss of self-tolerance and the development of autoimmunity, and not just the downstream consequence of autoreactive T_H_ cell activation (29). Strikingly, longitudinal studies have shown that disease-associated autoantibodies anticipate disease onset by years in multiple autoimmune disorders (30–33).

The BCR is basically a membrane-bound immunoglobulin (34). Upon ligation, the associated CD79a and b are phosphorylated by the Src-family kinase (SFK) Lyn (35–37) leading to Syk binding and phosphorylation (38). Bruton’s tyrosine kinase (Btk) is activated, which phosphorylates phospholipase C-gamma 2 (PLCγ2), leading to the cleavage of phosphoinositide PI (4, 5)P2, and the release of IP3 and DAG (39, 40), leading to Ca^2+^-release from the endoplasmic reticulum into the cytoplasm. This initial calcium release triggers the activation of calcium-release-activated (CRAC) channels, which allow the further influx of extracellular Ca^2+^ and prolonged Ca^2+^ flux constituting the plateau phase of the activation curve (41, 42). The Ca^2+^ flux response is a central activation pathway and its alterations have been linked to autoimmune disorders and B cell malignancies (43, 44).

Genome-wide association studies (GWAS) have identified a large number of autoimmunity promoting polymorphisms, many of which affect the signaling pathway of the BCR (43–49). Enhanced Ca^2+^ flux in B cells has been shown to promote autoimmunity (44). For example, polymorphisms in PTPN22 (Protein tyrosine phosphatase, non-receptor type 22) which are known to be associated with HT and other autoimmune disorders cause altered Ca^2+^ flux upon BCR ligation and B cell-restricted expression of risk variants is enough to initiate autoimmunity (29, 50–54).

There is a growing body of evidence directing our attention to the pivotal role of altered BCR signaling in driving autoimmunity by skewing the naive BCR repertoire, promoting the activation of autoreactive B cell clones both in a T cell- dependent and independent fashion, facilitating the formation of spontaneous autoreactive germinal centers and initiating the break in T cell tolerance. Evidence indicates, that B cells also play the crucial role of antigen presentation to T cells (29).

There is a considerable diversity in the Ca^2+^ flux patterns of B cells, suggesting that this mechanism is capable of encoding and transducing differences in the BCR signaling to distinct B cell subsets. Naive B cells express IgD and IgM and are negative for CD27. The CD19^+^ IgD^+^, CD27^-^ subpopulation also contains the newly formed, transitional B cells. Following activation, some B cells differentiate into memory B cells, express CD27 and re-enter the circulation. Peripheral memory B cells which still express IgD and IgM are termed ‘non-switched memory cells’, whereas those that undergo Ig-class switching and express IgG and further Ig subtypes are known as ‘switched memory’ cells. Certain memory cells lose their CD27 expression and become IgD^-^, CD27^-^ ‘double-negative’ memory B cells, which are non-dividing, inflammatory cytokine-producing cells that could play a role in autoimmunity (55–57). Recent studies suggest that B lymphocytes that express CD25 might represent a functionally distinct subset of cells, with higher levels of surface Ig expression and enhanced antigen-presenting ability (58).

The functional alterations of B cell subsets have never been investigated in HT before. We hypothesized that altered BCR signaling could play a role in thyroid autoimmunity and related infertility. Therefore, we have developed a flow cytometry method which enables the monitoring of the BCR ligation induced Ca^2+^ flux in the selected peripheral blood B lymphocyte subsets simultaneously. We also aimed to assess the effect of levothyroxine on the Ca^2+^ flux kinetics of B cells by sampling hypothyroid patients before and after treatment.

## Materials and methods

### Study design

Our pre-defined hypothesis was that altered BCR signaling could play a role in thyroid autoimmunity and related infertility. We designed a controlled laboratory experiment and enrolled ATA+, infertile, euthyroid patients; hypothyroid, ATA+ patients; and healthy volunteers. We developed a flow cytometry method which enables the monitoring of the BCR ligation induced Ca^2+^ flux in the selected peripheral blood B lymphocyte subsets simultaneously. The objective of the research was to compare the Ca^2+^ flux response of circulating B lymphocyte subsets in ATA+, infertile, euthyroid patients (1), hypothyroid, ATA+ patients before (2) and after levothyroxine treatment (3), and age-matched healthy controls (4). The secondary objective was to assess the effect of levothyroxine on the Ca^2+^ flux kinetics of B cells.

### Sample size

The number of replicates necessary to show the differences between study groups were defined by power analysis using the AUC parameter of the kinetic data from the first 3 samples of each group. The number of replicates necessary was determined as 12. On each measurement day one sample was collected. The number of participants in patient groups is higher, because all eligible patients were enrolled during the enrollment period (from March of 2019 to March of 2021). Extra patients were included to compensate for the potential samples lost due to unforeseeable circumstances such as technical error, secondary autoimmune disorder, COVID-19 infection, unplanned pregnancy. Data were only excluded if the kinetic curves could not be correctly fitted due to too low event number. Outliers were identified using the two-sided Grubbs’ test, but only extreme outliers were removed from the analysis.

### Human subjects

Our study was approved by the Regional Research Ethics Committee of the Medical Center, University of Pécs (RIKEB 5913/2015), and written informed consent was obtained from all participants. The study adhered to the tenets of the most recent revision of the Declaration of Helsinki.

### Patients

Fifteen patients with hypothyroidism due to Hashimoto’s thyroiditis were enrolled in the hypothyroid arm of the study. Clinical inclusion and exclusion criteria are summarized in **Table 1**. The TSH value of all patients was above the upper limit of the normal range (> 4.2 mU/l). Twelve patients had anti-TG antibody positivity (defined as anti-TG antibody level above the upper limit of the normal range, > 40 IU/ml), 13 had anti-TPO antibody positivity (defined as anti-TPO antibody level above the upper limit of the normal range, > 35 IU/ml), and all patients had signs of autoimmune thyroiditis on the ultrasound (see **Table 2**).The first samples were collected prior to any thyroid replacement therapy (H1), and the second sampling was done after the normalization of TSH, defined as TSH within the normal range, 0.270 - 4.2 mU/l (H2). One of the first samples had to be excluded due to a technical error. The second sample could not be collected from three patients, in two cases due to the development of a second autoimmune disease over time and in one case due to an unplanned pregnancy.

**Table 1:** Clinical inclusion and exclusion criteria for patient groups.

HC	Inclusion criteria	Exclusion criteria
Healthy control group	Healthy women	History of autoimmune disease
	Age 20-40 years	Abnormal TSH
	Normal cycle	Anti-thyroid antibody positivity
	Samples were collected between the 1^st^ and 5^th^ day of the cycle	History of infertility
		History of any chronic disease, genetic abnormality
		
**H1**		
Hashimoto hypothyroid group	Women	History of second autoimmune disease (autoimmune polyglandular syndrome)
	Age 20-40 years	
	Normal cycle	
	TSH > 4.2 mU/l	History of infertility
	Anti-thyroid antibody positivity (at least 1)	Other known cause of hypothyroidism
	Not treated with Levothyroxine	Thyroid nodule requiring surgery or radioiodine therapy
	Samples were collected between the 1^st^ and 5^th^ day of the cycle	History of other chronic disease, genetic abnormality
		
**H2**		
Follow-up sample from the same patients (H1) after the normalization of TSH due to levothyroxine therapy	TSH < 4.2 mU/l	Second autoimmune disease during follow-up (autoimmune polyglandular syndrome)
	Samples were collected between the 1^st^ and 5^th^ day of the cycle	Unplanned pregnancy before the 2^nd^ sample could be collected
	Levothyroxine therapy required to maintain normal value of TSH	
**HIE**		
Hashimoto infertile, euthyroid patients	Women	History of other autoimmune, chronic disease or genetic abnormality
	Age 20-40 years	Andrological abnormalities in the partner
	Normal cycle	History of endometriosis prior to enrollment
	Normal ovulation confirmed by ultrasound and elevated progesterone (> 10 nmol/l) on cycle day 21.	Tubal infertility
	Samples were collected between the 1^st^ and 5^th^ day of the cycle prior to assisted reproductive treatments	Anovulatory cycles
	Primary infertility in anamnesis: inability to conceive after at least 1 year of regular unprotected intercourse	Other known cause of infertility
	Applied for assisted reproductive care	Significant obesity (BMI > 35 kg/m^2^)
	TSH < 4.2 mU/l	Previous pregnancy (except first trimester miscarriage)
	Anti-thyroid antibody positivity (at least 1)	History of hypothyroidism
	No previous levothyroxine treatment	

TSH, Thyroid-stimulating hormone; BMI, body mass index.

**Table 2:** Clinical characteristics of patients.

	Healthy control (HC) (n = 12)	Hypothyroid before treatment (H1) (n = 15)	Hypothyroid after treatment (H2) (n = 12)	Euthyroid, ATA+, infertile (HIE) (n = 14)
Demographic data (mean + SD)				
Age (years)	29.7 (± 4.5)	31.6 (± 5.9)	31.9 (± 5.2)	31.2 ( ± 5.2)
BMI (kg/m2)	23.1 (± 5.1)	25.2 (± 5.7)		21.7 (± 2.8)
Laboratory parameters (median + IQR)				
TSH (mU/l)	1.63 (0.92 - 2.40)	6.81 (5.53 - 8.73)*	1.54 (0.60 - 2.27)	2.34 (1.62 - 3.30)
Anti-TPO (IU/ml)	9.85 (8.40 - 13.25)*	248.80 (70.00 - 388.90)	236.10 (102.90 - 374.60)	134.60 (17.00 - 203.10)*
Anti-TG (IU/ml)	12.35 (10.00 - 15.18)*	220.10 (42.90 - 318.80)	274.90 (58.53 - 451.20)	204.00 (16.78 - 355.30)
Estradiol (pmol/l)	141.50 (103.00 - 155.30)	120.00 (99.00 - 141.00)	149.00 (103.00 - 218.50)	147.00 (89.00 - 190.30)
Progesterone (nmol/l)	0.80 (0.50 - 1.28)	0.50 (0.30 - 0.90)	0.80 (0.53 - 2.30)	0.60 (0.48 - 1.00)
FSH (U/l)	6.70 (4.91 - 7.53)	6.40 (5.70 - 7.03)	6.33 (4.85 - 7.72)	6.95 (6.08 - 9.45)
LH (U/l)	4.00 (2.45 - 5.85)	3.65 (3.25 - 4.93)	4.15 (2.50 - 5.75)	3.65 (2.95 - 4.30)
Prolactin (ng/ml)	12.35 (10.07 - 19.53)	12.90 (10.07 - 17.42)	10.74 (8.15 - 11.85)	10.73 (7.78 - 17.52)
Testosterone (nmol/l)	1.00 (0.75 - 1.28)	1.10 (1.00 - 1.20)	1.17 (0.94 - 1.58)	0.95 (0.88 - 1.23)
Sex-hormone binding globulin (nmol/l)	82.65 (58.68 - 115.30)	62.60 (41.20 - 107.00)	46.85 (34.13 - 129.90)	68.40 (41.20 - 84.78)
CRP (mg/l)	0.60 (0.53 - 1.35)	2.25 (0.50 - 4.28)	2.75 (0.90 - 3.88)	1.20 (0.50 - 3.88)
Thyroid ultrasound findings				
Nodules (x/all)		7/15		1/14
Inhomogenity (x/all)		11/15		8/14
Hypoechogenic areas (x/all)		9/15		5/14
Hyper-vascularization (x/all)		8/15		6/14
Gynecological history				
Pregnancy (x/all)	3/12	5/15		2/14
Total number of pregnancies	7	8		3
Total number of miscarriages	2 (29%)	3 (37.5%)		3 (100%)
Total number of elective abortions	0	2 (25%)		0
Total number of term deliveries	5 (71%)	3 (37.5%)		0
Oral contraceptive in history (x/all)	11/12	13/15		9/14
Oral contraceptive at enrollment (x/all)	2/12	3/15		0/14
Allergy in history (x/all)	4/12	2/15		3/14

The laboratory results are from the day of enrollment. All patients were between the first and fifth day of the cycle. *p < 0.05 vs all other groups.

TSH, Thyroid-stimulating hormone; Anti-TPO, Thyroid Peroxidase Antigen; Anti-TG, Antithyroglobulin; FSH, Follicle stimulating hormone; LH, Luteinizing hormone; CRP, C-reactive protein; SD, Standard deviation; IQR, Interquartile range.

Fifteen anti-thyroid antibody positive, euthyroid patients were enrolled in the infertile arm of the study in whom immunological infertility could be assumed (Hashimoto’s thyroiditis, infertile, euthyroid group, HIE). Infertility was defined as inability to conceive after 1 year of regular unprotected intercourse. Twelve patients had anti-TG antibody positivity and 10 had anti-TPO antibody positivity. All patients were referred from an assisted reproduction program and samples were collected prior to any fertility treatments. The average time of infertility was 3.6 ± 2.7 years. The partners’ andrological examination showed normal sperm cell count, viability, and morphology. Estrogen and progesterone levels were normal throughout the cycle, and ovulation was confirmed by the elevated progesterone value and ultrasound examination. Tubal infertility was excluded by laparoscopic chromohydrotubation. All patients had primary infertility; no previous pregnancies (carried to term) were reported. Two patients had spontaneous early miscarriages prior to enrollment, but both met the inclusion criteria and conceived after over one year’s time. No other autoimmune disease had been diagnosed prior to the sample collection. Due to a technical error one sample could not be evaluated and therefore this patient was excluded from the analysis.

Twelve healthy age-matched controls (HC) were selected after screening for anti-thyroid antibodies and TSH. Routine laboratory parameters were also measured to exclude concomitant diseases. None of the healthy controls had any concomitant illnesses, any disorders in their menstrual cycle and none of them took any medication regularly.

All samples were collected between the 1st and the 5th day of the menstrual cycle to standardize the potential immunological effects of estrogen and progesterone. Patients had no infection at least 3 weeks prior to sampling, and CRP values were measured on the day of the blood draw. The patients had not been vaccinated in the 2 months before the sample collection. General medical and obstetric anamnesis, concomitant medication and illnesses, previous surgeries were recorded. The following laboratory parameters were measured on the day of the immunological sampling: TSH, anti-TPO, anti-TG, estradiol, progesterone, FSH, LH, prolactin, testosterone, sex-hormone binding globulin, CRP. The clinical characteristics of all participants are summarized in **Table 1**.

Our study was conducted in accordance with the Declaration of Helsinki. The research plan was approved by the local ethical board, the Regional Research Ethics Committee of the Medical Center, University of Pécs (number of approval: RIKEB 5913/2015). Consistently, written informed consent was obtained from all participants before any study procedures.

### Cell surface staining

Peripheral blood samples were collected from each participant (6x10 ml in lithium-heparin tubes, 3x6 ml in serum tubes). Peripheral blood mononuclear cells (PBMCs) were separated by Ficoll-Paque PLUS (GE Healthcare) gradient centrifugation. The cell suspension was washed twice in PBS and resuspended in RPMI 1640 containing 5% fetal bovine serum (FBS) (Sigma-Aldrich).

All measurements were performed on freshly prepared PBMCs. The times from sample acquisition to measurement were standardized throughout the entire protocol. PBMCs (8 x 10^6^ cells) were stained with the appropriate combination of fluorochrome-conjugated anti-human antibodies in 100 µl of media in order to differentiate B lymphocyte subsets: CD5 PerCP (clone UCHT2), CD19 PE (clone SJ25C1), IgD APC (clone IA6-2), CD25 BV421 (clone BC96) (all from Sony Biotechnology), CD27 PE-Cy7(clone M-T271, BioLegend). The cells were incubated with the antibodies for 30 minutes in the dark at room temperature (RT), according to the manufacturer’s instructions. Following labeling, samples were washed twice in RPMI.

### Loading with calcium indicator dye

Cytoplasmic free, ionized calcium level was detected by loading the cells with 5 μM Fluo-4- acetoxymethyl (AM) (Invitrogen, Thermo Fisher Scientific in DMSO, Sigma-Aldrich) supplemented with 20% (w/v) Pluronic F-127 (Sigma-Aldrich) for 15 minutes in the dark at RT. Cells were washed and then kept in RPMI medium supplemented with CaCl_2_ to a Ca^2+^ concentration of 1.8 mM. Cells were incubated in RT in the dark for a further 30 minutes to allow complete de-esterification of intracellular Fluo-4-AM esters. Each sample was measured directly after the de-esterification period.

### Flow cytometry measurements

Fluorescence data from each sample were measured and recorded for 17 minutes in a kinetic manner. Unstimulated controls were measured for the first 2 minutes for baseline acquisition, then B lymphocytes were activated by crosslinking the B cell receptor (BCR) with 10 µg/ml F(ab’)_2_ Fragment AffiniPure Goat Anti-Human IgG + IgM (H+L) (Jackson ImmunoResearch) and samples were measured for another 15 minutes. Flow cytometry measurements were performed using FACS Canto II (BD Biosciences).

### Analysis, controls, and reproducibility

In order to ensure flawless measurements and comparable experiments, cytometer setup and tracking (CS&T) measurement was carried out on every measurement day. Compensation controls were measured, and matrices were calculated specifically for each measurement day in FlowJo (V10.7.1.), using compensation beads (BD) for fluorochrome-conjugated antibodies and the test subject’s PBMCs for Fluo-4. Data were analyzed using FlowJo v10.7.1 (BD Biosciences). All gates were set in FlowJo, using Fluorescent Minus One (FMO) controls for each sample (**Supplementary Figure 1**).

Following gating, the compensated FCS data of each subpopulation were separately exported. Kinetic curves were calculated by FacsKin (https://facskin.bitbucket.io/) using an algorithm that fits a function to the median fluorescence intensities of the calcium indicator dye, Fluo-4. This allows the mathematical description and statistical comparison of flow cytometry acquired kinetic measurements. The calcium flux of B cells can be described by a double-logistic function, as the intracellular calcium level first increases, reaches a maximum level, then decreases and reaches a plateau. Each curve is described by the following parameters: starting value; time to reach the first 50% value, slope at first 50% value (ascending phase), time from first 50% to maximum, time to reach the maximum value, maximum value, time from maximum to second 50%, slope at second 50% value (descending phase), ending value, and area under the curve (AUC).

**Figure 1:**
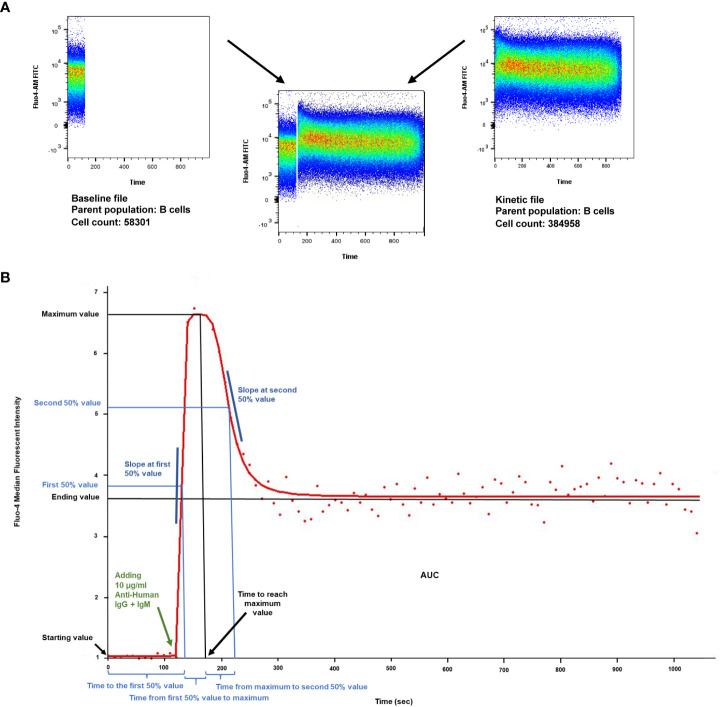
Calcium flux kinetic parameters. **(A)** Density plot of intracellular Ca^2+^ mobilization in human B cells in response to anti-IgG+M antibody stimulation after labeling with Fluo4- AM (analyzed by FlowJo v10.7.1). X axis: time, Y axis: Fluo4-AM (FITC). Unstimulated controls were acquired for the first 120 seconds for baseline, then B lymphocytes were activated by crosslinking the B cell receptor with 10 µg/ml F(ab’)_2_ Fragment AffiniPure Goat Anti-Human IgG + IgM (H+L). Activated samples were recorded for 15 minutes (900 s) in a kinetic manner. **(B)** Parameters of the dlogist+ (Double-logistic) kinetic function calculated by FacsKin. Example of a Ca^2+^ flux kinetic curve after anti-IgG+M activation. X axis: time, Y axis: relative parameter value: standardized based on the Fluo4- AM MFI (median fluorescent intensity). The kinetic curve is created based on the median values of the gated population. The biologically relevant parameters are the following: starting value, calculated as the limit of the function at -∞ (minus infinity) (standardized to 1); time to the first 50% value (s); slope at first 50% value, which is always positive and defines how much the intensity changes during 1 second (unit: int/s where int is the unit of the vertical axis); time from first 50% to maximum value (s), time to reach maximum value (s), maximum value (relative value); time from maximum to second 50% value (s); slope at second 50% value, always negative (unit: int/s); ending value, calculated as the limit of the function at +∞ (positive infinity), AUC: the area under curve from time point 0 to 1020 s (manually set to actual time of measurement).

The starting value is determined by the baseline calcium level of each sample and subset and every curve is standardized to start at 1. Therefore, measurements from different subsets, patients and days can be compared objectively. The maximum value of the Fluo-4 MFI correlates with the peak Ca^2+^ level following an activating stimulus. The ending value shows the MFI of the Fluo-4 at the end of the measurement period, which in a 15-minute measurement corresponds with the plateau phase of the activation curve. The time and slope parameters indicate how quickly the given cells are able to mobilize Ca^2+^ upon BCR stimulation. The area under the curve (AUC) value is the most sensitive, as it indicates the total capacity of cells to mobilize free Ca^2+^ into the cytoplasm. (**Figure 1**).

Various viability tests were performed. In a separate tube, a viability dye (Zombie NIR, from Biolegend) was added at the time of cell surface labeling to check viability of the cells prior to loading with Fluo-4. was also checked during gating, as Fluo-4+ cells were gated. As only live cells with intact membrane can be loaded with Fluo-4 we used this method to exclude cells that might have been damaged during labeling. All samples had over 95% viability within the lymphocyte gate with both methods. After measuring each tube, trypan blue was used to check that the activation process did not lead to rapid cell death.

### Statistics

In order to detect the differences between the HC-H2, HC-H1, HC-HIE, H2-HIE, H1-HIE patient groups we used ordinary two-way ANOVA and Tukey’s *post hoc* test. Differences between the H1 and H2 groups were assessed by a mixed-effects analysis and the Sidak’s multiple comparisons test due to repeated sampling and randomly missing values. Levels of comparisons are the follows; (1) between the mean of the patient groups when completely ignoring the grouping of the cells, (2) between the mean of the patient groups within the cell populations and subpopulations, (3) between the mean of cell populations and subpopulations within the patient groups. Statistical analysis was carried out in Prism 8 (GraphPad Software, San Diego, CA, USA).

## Results

### Different naive and memory B cell ratio in levothyroxine treated and ATA+, euthyroid, infertile patients

**Figure 2:**
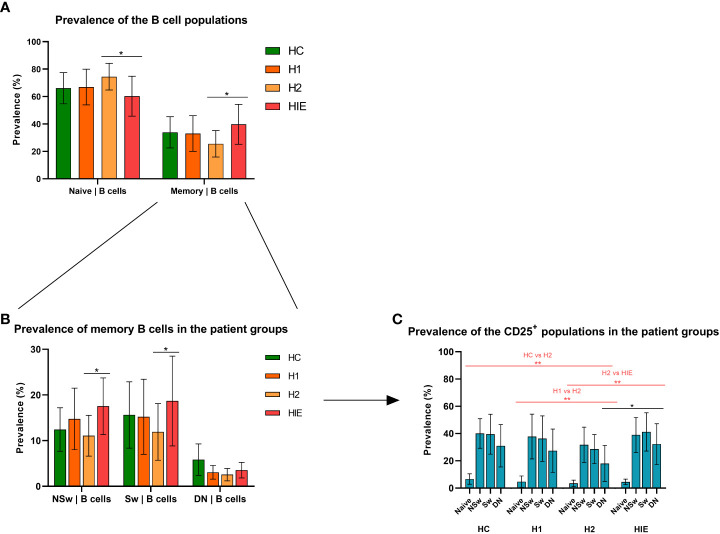
Different naive and memory B cell ratio in levothyroxine treated and ATA+, euthyroid, infertile patients Prevalence of naive and memory B cell subsets **(A)**, B cell memory subpopulations **(B)** and CD25^+^ B cell subpopulations **(C)** HC: healthy controls (n=12); HT: Hashimoto’s thyroiditis, H1: hypothyroid patients with HT before treatment (n=14); H2: euthyroid patients with HT, after levothyroxine-treatment, (n=12); HIE: HT, infertile, euthyroid patients (n=14); NSw: non-switched; Sw: switched, DN: double negative memory B cells. Differences between the groups and within the B cell subpopulations were assessed with two-way ANOVA and Tukey’s *post hoc* test except H1-H2 comparisons where a mixed-effects analysis and the Sidak’s multiple comparisons tests were used because these groups represent the same individuals in a repeated sampling before and during treatment. Bars represent the mean ± standard deviation (SD) of percentages of lymphocyte subpopulation in each group. *p < 0.05, **p < 0.01.

The prevalence of B and T lymphocytes was comparable in the different patient groups. Within the B cell subpopulations, the prevalence of naive B cells was higher in the euthyroid, levothyroxine treated Hashimoto (H2) group compared to the ATA+, infertile, euthyroid (HIE) group (p=0.0230), while H2 patients had significantly lower number of memory cells compared to HIE patients (p=0.0230). **Figure 2A**).

The prevalence of the non-switched memory (NSw) (p=0.0289) and the class-switched memory (Sw, p=0.0196) B cells was significantly higher in the HIE group compared to the H2 patients (**Figure 2B**).

### The CD25-expressing B lymphocyte compartment is smaller in levothyroxine treated patients

The prevalence of CD25 expressing cells within all B cell subpopulations did not differ among the healthy control (HC), the hypothyroid, untreated (H1) and HIE patients, but was significantly lower in the H2 group (*vs* HC p=0.0008, *vs* H1 p=0.0021, *vs* HIE p=0.0003). The prevalence of CD25^+^ CD27^-^ IgD^-^, double negative (DN) memory cells was lower in the H2 group compared to the HIE group (p=0.0121) (**Figure 2C**).

### Basal calcium level is elevated in B cells from ATA+, euthyroid, infertile patients

**Figure 3:**
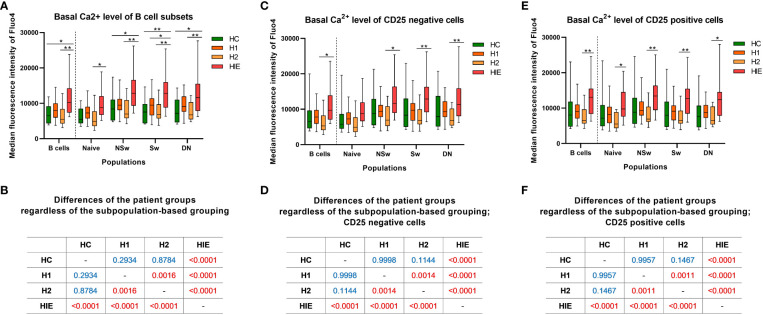
Basal calcium level is elevated in B cells from ATA+, euthyroid, infertile patients Baseline median fluorescence intensity of Fluo4 in B cell subpopulations **(A)**, CD25- **(C)** and CD25+ **(E)** B cells. Cross tabulation shows the p values when comparing patient groups without the subpopulation-based grouping of B cells **(B)**, CD25- **(D)** and CD25+ cells **(F)**. HC: healthy controls (n=12); HT: Hashimoto’s thyroiditis, H1: hypothyroid patients with HT before treatment (n=14); H2: euthyroid patients with HT, after levothyroxine-treatment, (n=12); HIE: HT, infertile, euthyroid patients (n=14); NSw: non-switched; Sw: switched, DN: double negative memory B cells. Differences between the groups and within the B cell subpopulations were assessed with two-way ANOVA and Tukey’s *post hoc* test except H1-H2 comparisons, where a mixed-effects analysis and the Sidak’s multiple comparisons test were used because these groups represent the same individuals in a repeated sampling before and during treatment. Data are presented as follows: middle line represents the median, dot represents the mean, box: interquartile range, whiskers: min-max. *p < 0.05, **p < 0.01; Significant p values are red, non-significant p values are written in blue.

The baseline Ca^2+^ level (indicated by the median fluorescence intensity (MFI) of Fluo-4) of total B cells from ATA+, infertile, euthyroid patients (HIE) was higher compared to the other groups [*vs* HC (p=0.0186), *vs* H2 (p=0.0053); (**Figure 3A**)].

The baseline Fluo-4 MFI of naive B cells from HIE patients was higher compared to the H2 group (p=0.0320). Similar, more pronounced alterations were present in memory B cell subsets. The NSw memory cells of HIE patients showed significantly higher baseline Fluo-4 MFI compared to the HC (p=0.0168) and H2 (p=0.0017) groups. The baseline Fluo-4 MFI of Sw memory cells was higher in the HIE group in comparison with the HC (p=0.0067), the H1 (p=0.0437) and the H2 (p=0.0022) groups. The DN memory subset of the HIE group also showed elevated basal Fluo-4 MFI compared to the HC (p=0.0201) and the H2 (p=0.0054) groups. (**Figure 3A**).

When investigating the differences in the basal Fluo-4 level between the patient groups, regardless of the subpopulation-based grouping, significant differences were found in the following comparisons: HC<HIE (p<0.0001), H1<HIE (p<0.0001), H2<HIE (p<0.0001), H1>H2 (p=0.0016). (**Figure 3B**).

When dividing B cells and subsets based on their CD25 expression, similar alterations were present in both CD25 negative (CD25^-^) and CD25 positive (CD25^+^) B cell subsets. In the case of CD25^-^ B cells, only the memory subsets showed higher baseline Fluo-4 MFI in the HIE group compared to the H2 group (NSw p=0.0123, Sw p=0.0045, DN p=0.0066). (**Figures 3C–F**).

Levothyroxine treatment decreased the baseline Ca^2+^ level, although neither pre- nor post-treatment values differed from the healthy control group (p=0.0011). This alteration is not only present in the CD25^+^ cells, but also in the CD25^-^ fraction of B cells. (**Figures 3B, D, F**).

### Naive B cells show higher Ca^2+^ signal upon BCR stimulation in ATA+ euthyroid, infertile patients

**Figure 4:**
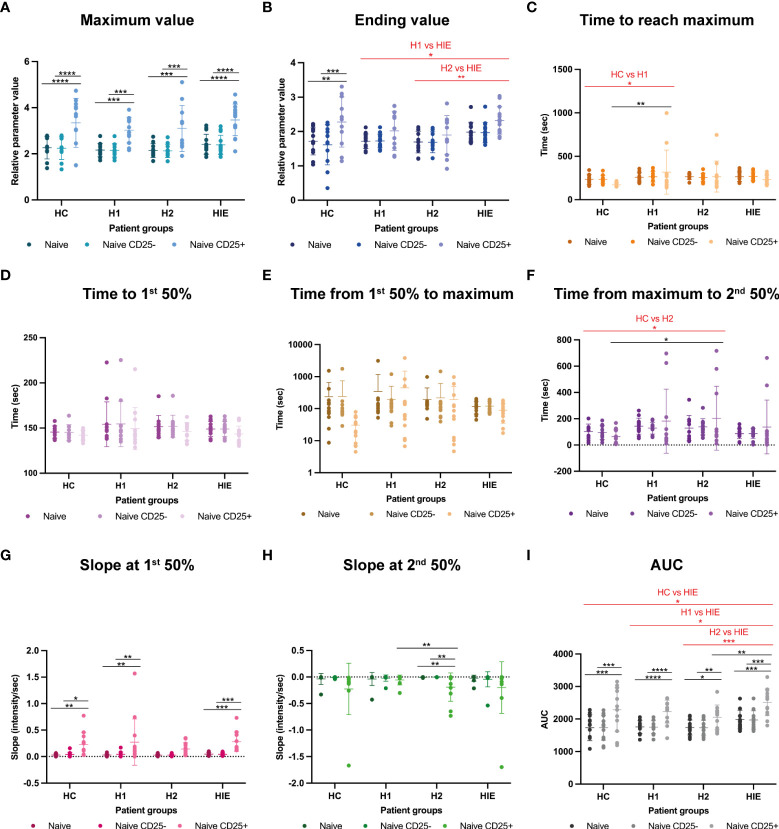
Naive B cells show higher Ca^2+^ signal upon BCR stimulation in ATA+ euthyroid, infertile patients Calcium flux kinetic data of naive B, naive CD25- and naive CD25+ B cells **(A)** maximum value, **(B)** ending value, **(C)** time to reach maximum, **(D)** time to first 50% value, **(E)** time from first 50% to maximum, **(F)** time from maximum to second 50% value, **(G)** slope at the first 50% value, **(H)** slope at second 50% value, **(I)** area under curve (AUC) value. HC: healthy controls (n=12); HT: Hashimoto’s thyroiditis, H1: hypothyroid patients with HT before treatment (n=14); H2: euthyroid patients with HT, after levothyroxine-treatment, (n=12); HIE: HT, infertile, euthyroid patients (n=14); NSw: non-switched; Sw: switched, DN: double negative memory B cells. The kinetic data were analyzed by the FACSKin software. Differences between the groups were assessed with two-way ANOVA and Tukey’s *post hoc* test except H1-H2 comparisons where a mixed-effects analysis and the Tukey’s multiple comparisons test were used because these groups represent the same individuals in a repeated sampling before and during treatment. Data are depicted as individual values, middle line represents the mean, whiskers are set to standard deviation (SD) *p < 0.05, **p < 0.01, ***p < 0.001, ****p < 0.0001.

Then we compared the BCR stimulation induced Ca^2+^ flux properties in CD25^+^ and CD25^-^ naive B cells of the different patient groups. The maximum Fluo-4 MFI (peak of the Ca^2+^ signal) of CD25^+^ naive B cells was significantly higher compared to the total and CD25^-^ naive B cell populations within all patient groups. No significant differences were present between the patient groups. (**Figure 4A**).

In contrast to the Max value, the ending Fluo-4 MFI was only higher in the CD25^+^ naive cells compared to the CD25^-^ cells in the HC group. HIE patients had higher Ending Fluo-4 MFI values compared to H1 (p=0.0269) and H2 (p=0.0050) groups. (**Figure 4B**).

The CD25^+^ naive cells of the untreated H1 patients required significantly more time to reach the maximum Fluo-4 MFI value of the activation compared to the HC group (p=0.0028). (**Figure 4C**). Time to 1st 50% and Time from 1st 50% to maximum parameters were comparable between the groups (**Figures D, E**).

In the descending phase of the activation, the CD25^+^ cells of the H2 patients required more time to reach the second 50% value of the curve, indicating that they need more time to reduce the intracellular free Ca^2+^ concentration than those of the HC group (p=0.0451). (**Figure 4F**).

The ascending (1^st^) slope was significantly steeper in CD25^+^ cells in the HC, H1 and HIE groups compared to CD25^-^ cells and to the whole naive population. (**Figure 4G**).

In the H2 group, the slope of the descending phase of the activation was significantly steeper in CD25^+^ cells compared to CD25^-^ cells (p=0.0024) and to the whole naive population (p=0.0024). Furthermore, naive CD25^+^ cells from H2 patients showed significantly steeper 2^nd^ slope values compared to naive CD25^+^ cells from the H1 group (p=0.0475). (**Figure 4H**).

The AUC of CD25^+^ naive B cells was higher in every patient group compared to the CD25^-^ and total naive cells. The AUC value of the naive B cell population of HIE patients was higher compared to the HC (p=0.0200), to the H1 (p=0.0130) and to the H2 (p=0.0009) groups. The CD25^+^ naive B cells of HIE patients had higher AUC values than those of the H2 group (p=0.0070). (**Figure 4I**).

### Memory B cells of ATA+, euthyroid, infertile patients have enhanced Ca^2+^ flux upon BCR activation

**Figure 5:**
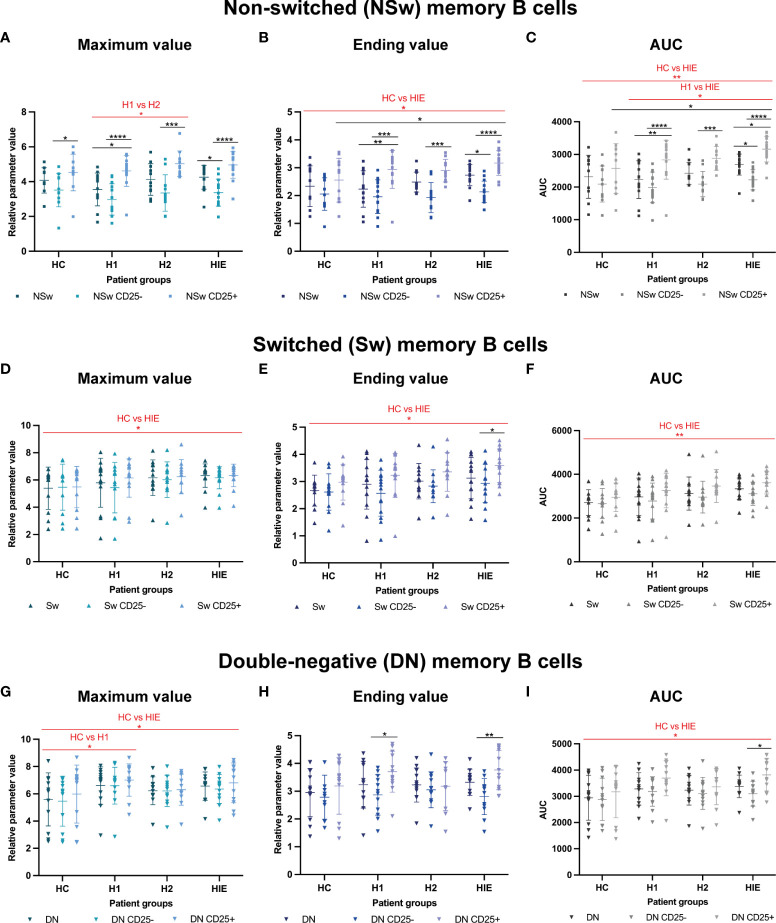
Memory B cells of ATA+, euthyroid, infertile patients have increased calcium flux upon BCR activation Selected calcium flux kinetic data of memory B cell subsets: Non-switched (NSw) memory B cells, CD25- and CD25+ NSw cells: **(A)** maximum value, **(B)** ending value, **(C)** area under curve (AUC) value. Immunoglobulin class-switched (Sw) memory B cells, CD25- and CD25+ Sw cells: **(D)** maximum value, **(E)** ending value, **(F)** area under curve (AUC) value. CD27-, IgD- double-negative (DN) memory B cells, CD25- and CD25+ DN cells: **(G)** maximum value, **(H)** ending value, **(I)** area under curve (AUC) value. HC: healthy controls (n=12); HT: Hashimoto’s thyroiditis, H1: hypothyroid patients with HT before treatment (n=14); H2: euthyroid patients with HT, after levothyroxine-treatment, (n=12); HIE: HT, infertile, euthyroid patients (n=14). The kinetic data were analyzed by the FACSKin software. Differences between the groups were assessed with two-way ANOVA and Tukey’s *post hoc* test except H1-H2 comparisons where a mixed-effects analysis and the Tukey’s multiple comparisons test were used because these groups represent the same individuals in a repeated sampling before and during treatment. Data are depicted as individual values, middle line represents the mean, whiskers are set to standard deviation (SD). *p < 0.05, **p < 0.01, ***p < 0.001, ****p < 0.0001.

Similarly, to the naive B cells, CD25^+^ NSw memory B cells had higher Max values compared to the whole NSw subpopulation and CD25^-^ NSw cells. When comparing the patient groups, regardless of the CD25-based grouping, we found that the maximum Fluo4 MFI was higher after levothyroxine treatment (H2) compared to before treatment values (H1, p=0.0385). (**Figure 5A**).

The ending Fluo-4 MFI of CD25^+^ NSw memory cells was also higher in all patient groups (H1, H2, HIE) compared to the total NSw population and the CD25^-^ NSw cells. The HIE group had a higher ending values than the HC group (p=0.0308) and CD25^+^ NSw cells of HIE patients had higher ending values than CD25^+^ NSw cells of HC (p=0.0357). (**Figure 5B**) The AUC values of the CD25^+^ NSw memory cells were significantly higher than those of CD25^-^ NSw cells in all three (H1, H2, HIE) patient groups. The CD25^+^ NSw cells of HIE patients had exceptionally high AUC values, significantly higher compared to the CD25^+^ NSw cells of healthy controls. When looking at patient groups regardless of the CD25 expression-based grouping, the difference between the AUC values of NSw cells from HIE patients was not only higher compared to healthy controls (HC, p=0.0097), but also compared to hypothyroid patients (H1, p=0.0121). (**Figure 5C**) The CD25^+^ NSw cells also reacted faster than CD25^-^ NSw cells in all groups. Significant differences could be detected in pattern of activation between the HC and H1; and the HC and HIE groups. Further time and slope parameters of NSw memory cells are presented in **Supplementary Figure 2**.

The Sw memory cells of HIE patients showed higher Maximum, Ending and AUC values compared to the HC group (p=0.0487, p=0.0282 and p=0.0015 respectively). (**Figures 5D-F**) The CD25^+^ compartment of the Sw memory cells also had higher Ending value compared to the CD25^-^ cells in HIE patients (p=0.0479). (**Figure 5E**) The Sw cells of H1 patients required more time to reach the maximum value compared to HC (p=0.0044), whereas the Sw cells from the same patients after treatment (H2) needed less time compared H1 (p=0.0309). (**Supplementary Figure 3**).

The DN memory cells of both H1 and HIE patients showed higher maximum Fluo-4 MFI values compared to the healthy controls (p=0.0102 and p=0.0449, respectively). (**Figure 5G**) CD25^+^ DN memory cells showed higher ending Fluo4 MFI compared to CD25^-^ cells in the H1 (p=0.0202) and in the HIE (p=0.0067) groups. (**Figure 5H**) CD25^+^ DN cells had higher AUC values compared to CD25^-^ DN cells in the infertile (HIE) group. DN memory B cells from HIE patients had an overall higher AUC compared to healthy controls (HC, p=0.0359). (**Figure 5I**) DN memory cells of HIE patients reacted slightly faster (higher 1^st^ slope) to BCR stimulation compared to controls (HC, p=0.0467). (**Supplementary Figure 4**)

## Discussion

In this study we investigated the Ca^2+^ flux of B cell subsets in response to BCR ligation in infertile, euthyroid, ATA+ women; hypothyroid, ATA+ patients before and after levothyroxine treatment and age-matched healthy controls. The functional alterations of B lymphocytes have never been investigated in Hashimoto’s thyroiditis and infertility before. Signaling *via* the BCR is the ‘master regulator’ of most aspects of immunological fate in B cells including positive and negative selection, central and peripheral tolerance, activation and function (29). Ca^2+^ flux serves as a central pathway for encoding and transducing differences in BCR signaling with significant biological and pathological consequences (43, 44). The BCR signaling is modified by synergizing signals from several receptors, such as the B cell-activating factor receptor (BAFFR), toll-like receptors (TLR) 7 and 9 and CD40 (45). Emerging evidence indicates that even modest alterations in BCR signaling are sufficient to facilitate and maintain autoimmunity in a B cell-intrinsic manner *via* several distinct mechanisms (29).

Altered signaling downstream of the BCR and synergizing receptors can lead to the skewing of the naive BCR repertoire resulting in an increased prevalence of autoreactive clones (59–63). However, this alone is not enough for the development of an autoimmune disease, as up to 40% of newly formed B cells exhibit autoreactivity in healthy individuals as well (64, 65). The critical step in the loss of tolerance is the activation of autoreactive B cell clones. Evidence indicates that during an autoimmune disorder autoantibody-producing B cells can be formed both in the germinal center and *via* the extra-follicular pathway. Remarkably, under specific circumstances newly formed B cells are able to by-pass the germinal center yet still go through somatic-hypermutation and antibody class-switching and differentiate into short lived plasmablasts in the periphery (66). An infection could be an important permissive circumstance, as increased BAFF and type I IFN production along with the presence of pathogen-derived antigens, which connect to the TLRs can create the opportunity for newly formed B cells to activate and go through class switching. Among these activated B cells a higher prevalence of self-reactive clones may initiate autoantibody production (64).

Recent studies have demonstrated that activated autoreactive B cells are able to direct the formation of spontaneous GCs and facilitate the break in T cell tolerance by recruiting cognate T_FH_ cells (29). It is intriguing to assume that B cells may be the ones that play the initial role of antigen presentation, providing the crucial activating signals for autoreactive T cells (29). In this regard, the CD25^+^ B cells may be especially interesting (58). In line with previous findings, suggesting that CD25^+^ B cells represent a functionally different group (58), we found enhanced responsivity to BCR stimulation in CD25^+^ B cells compared to CD25^-^ cells within the naive and non-switched subsets in healthy controls as well as all patient populations.

The B cells of infertile, ATA+ euthyroid patients showed higher basal level of Ca^2+^ and hyperresponsivity to BCR ligation, indicated by the altered Ca^2+^ flux kinetic parameters compared to healthy controls and hypothyroid patients. Interestingly, the elevated basal Ca^2+^ level was not only present in the CD25^+^ compartment of B cell subsets, but also the CD25^-^ compartment. The naive B cells of infertile patients had enhanced Ca^2+^ flux response compared to the healthy control group, which is especially interesting knowing that immature B cell clones that have enhanced response to BCR stimulation have an advantage during the positive selection. In experimental settings a slight increase in the BCR signaling strength was sufficient to promote autoimmunity in a B cell intrinsic manner (29). Memory subsets of infertile, ATA+ patients also showed BCR-hyperreactivity compared to healthy controls.

Surprisingly, we only found minimal differences between hypothyroid patients and healthy controls. However, we observed differences between the hypothyroid and the ATA+, infertile, euthyroid patient groups. The overall basal Ca^2+^ level of B cells of infertile patients was higher than that of hypothyroid patients. However, the differences in the Ca^2+^ flux kinetics between the euthyroid infertile and the hypothyroid group were only present in the naive and non-switched memory subsets. The naive subset had higher Ca^2+^ levels at the end of the measurement and both the naive and the non-switched memory cells had higher AUC in the infertile group compared to the hypothyroid group. The switched and double-negative memory subsets on the other hand showed similar Ca^2+^ flux characteristics in these two patient populations. The double-negative memory cells for example had a similarly elevated Max value in both the hypothyroid and the euthyroid, infertile group compared to healthy controls.

These findings indicate elevated basal Ca^2+^ levels and enhanced Ca^2+^ flux response in ATA+, euthyroid, infertile patients but not in hypothyroid patients. Two hypotheses could explain these findings, first, that these two patient populations represent different stages of thyroid disease, which could impact the characteristics of the autoimmune response. The ATA+, infertile, euthyroid patients still have maintained thyroid function, thus the observed alterations could be characteristics of an earlier stage of thyroid autoimmunity. In the hypothyroid group, the signs of thyroid tissue damage are evident on the thyroid ultrasound, and they have decreased thyroid function, indicating years of ongoing autoimmune inflammation. This would support the notion that the loss of tolerance for thyroid antigens is initiated in the periphery rather than in the thyroid gland itself. Second, the key difference between the H1 and HIE groups is the presence of infertility. In this context, the enhanced Ca^2+^ flux response of B cells may be a result of systemic immune dysfunction, which could be the common underlying cause of both infertility and HT. Overall, these data support the role of enhanced BCR-induced Ca^2+^ flux in the early phase of thyroid autoimmunity and infertility and lead us to believe that further investigating of this population is key to understanding the connection between Hashimoto’s thyroiditis and infertility.

To investigate the direct effect of hypothyroidism on B cell function, we evaluated the hypothyroid patients following levothyroxine replacement. After the normalization of TSH levels, the basal Ca^2+^ levels of these patients decreased compared to pre-treatment, although neither the pre- nor post-treatment values differed from the healthy controls. More interestingly, levothyroxine treatment decreased the prevalence of CD25^+^ B cells, which are presumed to play an important role in peripheral antigen presentation to T cells (58). One reason for this could be a lower autoantigen release due to decreased metabolic activity of thyroid follicles or it may be a direct effect of levothyroxine. This is a potential mechanism how the levothyroxine treatment of hypothyroidism could decrease the progression rate of autoimmune thyroid destruction.

Interestingly, we found differences between the two euthyroid patient groups (H2 and HIE) as well. The prevalence of naive B cells was lower, while the prevalence of memory B cells was higher in ATA+, infertile, euthyroid patients compared to levothyroxine-treated, euthyroid patients. The prevalence of CD25^+^ B cells was lower in the levothyroxine treated group, specifically in the double-negative memory subset. The basal Ca^2+^ level was higher in infertile patients in all B cells and subsets. The naive cells of infertile patients had higher intracellular Ca^2+^ level at the end of the measurement and the whole naive population and the CD25^+^ naive cells had higher overall Ca^2+^ flux (AUC) compared to the levothyroxine treated group. As both groups were in a euthyroid state, it seems that thyroid function on its own does not determine the Ca^2+^ flux response of peripheral B cells.

The greatest strength of this work is that utilizing this novel flow cytometry method enables us to describe the subtle alterations of the activation response of selected B lymphocyte subsets in real time. This gives us information about the function of B cell subsets and allows the objective comparison of subsets, as each receives the exact same activating stimulus. The alterations of slope and time parameters did not appear relevant in thyroid autoimmunity, however, to adhere to the rules of accurate scientific reporting we decided to present these parameters as well. The most important limitation of this study is that due to the very strict inclusion criteria, the highly specialized measurement method and the COVID-19 pandemic, the number of patients is limited. Therefore, these results have to be verified on larger patient populations as well. Although the autoimmune thyroid inflammation takes place in the thyroid gland itself and therefore investigating peripheral B cells has limitations, the thyroid-specific memory B lymphocytes can re-enter the circulation and reflect the ongoing autoimmune process. More importantly, as infertility suggests a systemic immune disorder, investigating peripheral B cell subsets is highly relevant in the question of how HT and infertility are related.

In conclusion, we were able to show systemic BCR-hyperresponsivity in ATA+, infertile, euthyroid patients, which was not present in hypothyroid patients. This supports the role of altered Ca^2+^ flux of B cells in the early phase of thyroid autoimmunity and infertility. We therefore suggest a paradigm shift, where B cells could play a role in the development of thyroid autoimmunity and infertility rather than being just by-standers in the inflammatory response. This could also mean that the thyroid gland is just one of the targets of autoreactive B cells and autoimmune thyroiditis might not be the underlying cause of infertility. However, further investigations are necessary to understand the association of Hashimoto’s thyroiditis and infertility. Levothyroxine treatment led to a subtle decrease in the prevalence of CD25^+^ B cells and lower basal Ca^2+^ levels compared to pre-treatment, which indicate that levothyroxine may have a slight effect on the progression of autoimmune inflammation. However, as differences were present between the two euthyroid groups, thyroid function alone is unlikely to determine the function of B cells.

## Data availability statement

The original contributions presented in the study are included in the article/**Supplementary Materials**. Further inquiries can be directed to AB at bajnok.panni@gmail.com.

## Ethics statement

This study was reviewed and approved by Regional Research Ethics Committee of the Medical Center, University of Pécs (RIKEB 5913/2015). The patients/participants provided their written informed consent to participate in this study.

## Author contributions

Conceptualization and experiment design TS-L, AB, VT, TB, and EM. Calcium-flux methodology AB, TS-L and VT. FacsKin Software development AK. Formal Analysis VT and JN. Investigation TS-L, AB and JN. Resources EM, TB and TK. Patient enrollment AB, EM, AV, KK and SP. Patient data acquisition GP-D. Writing – original draft, AB, TS-L, VT and EM. Writing – Review and Editing TB, TK and EM. Visualization, TS-L, VT, JN and GP-D. Supervision, EM and TB. Funding Acquisition, EM, TB and TK. All authors contributed to the article and approved the submitted version.

## Funding

This research was funded by the PTE ÁOK-KA grant (No: KA-2019-28), the Thematic Excellence Program 2020 - Institutional Excellence Sub-program of the Ministry for Innovation and Technology in Hungary, within the framework of the second thematic program of the University of Pécs and the European Union and co-financed by the European Social Fund: Comprehensive Development for Implementing Smart Specialization Strategies at the University of Pécs (EFOP-3.6.1.-16-2016-00004). This work was supported by the National Research, Development and Innovation Fund of Hungary, financed under the TKP-2021-EGA-10 funding scheme.

## Acknowledgments

The research was performed in collaboration with the Flow Cytometry Core Facility at the Szentágothai Research Centre of the University of Pécs. The present scientific publication is part of the Hungarian National Laboratory on Reproduction. The authors would like to thank Diána Simon for her contribution to the initial study concept.

## Conflict of interest

The authors declare that the research was conducted in the absence of any commercial or financial relationships that could be construed as a potential conflict of interest.

## Publisher’s note

All claims expressed in this article are solely those of the authors and do not necessarily represent those of their affiliated organizations, or those of the publisher, the editors and the reviewers. Any product that may be evaluated in this article, or claim that may be made by its manufacturer, is not guaranteed or endorsed by the publisher.

## References

[1] Pearce EN Farwell AP Braverman LE. Thyroiditis. New Engl J Med (2003) 348(26):2646–55. doi: 10.1056/NEJMra021194 12826640

[2] Hollowell JG Staehling NW Flanders WD Hannon WH Gunter EW Spencer CA. T(4), and thyroid antibodies in the united states population (1988 to 1994): National health and nutrition examination survey (NHANES III). J Clin Endocrinol Metab (2002) 87(2):489–99. doi: 10.1210/jcem.87.2.8182 11836274

[3] Vanderpump MP Tunbridge WM French JM Appleton D Bates D Clark F. The incidence of thyroid disorders in the community: a twenty-year follow-up of the whickham survey. Clin Endocrinol (Oxf) (1995) 43(1):55–68. doi: 10.1111/j.1365-2265.1995.tb01894.x 7641412

[4] Pyzik A Grywalska E Matyjaszek-Matuszek B Roliński J. Immune disorders in hashimoto’s thyroiditis: What do we know so far? J Immunol Res (2015) 2015:979167. doi: 10.1155/2015/979167 26000316 PMC4426893

[5] Thangaratinam S Tan A Knox E Kilby MD Franklyn J Coomarasamy A. Association between thyroid autoantibodies and miscarriage and preterm birth: meta-analysis of evidence. BMJ (Clinical Res ed) (2011) 342:d2616. doi: 10.1136/bmj.d2616 PMC3089879 21558126

[6] Zhong YP Ying Y Wu HT Zhou CQ Xu YW Wang Q. Relationship between antithyroid antibody and pregnancy outcome following *in vitro* fertilization and embryo transfer. Int J Med Sci (2012) 9(2):121–5. doi: 10.7150/ijms.3467 PMC3258552 22253557

[7] Gleicher N. Maternal autoimmunity and adverse pregnancy outcomes. J Autoimmunity (2014) 50:83–6. doi: 10.1016/j.jaut.2013.12.009 24461538

[8] Negro R Mangieri T Coppola L Presicce G Casavola EC Gismondi R. Levothyroxine treatment in thyroid peroxidase antibody-positive women undergoing assisted reproduction technologies: a prospective study. Hum Reprod (2005) 20(6):1529–33. doi: 10.1093/humrep/deh843 15878930

[9] Negro R Formoso G Mangieri T Pezzarossa A Dazzi D Hassan H. Levothyroxine treatment in euthyroid pregnant women with autoimmune thyroid disease: effects on obstetrical complications. J Clin Endocrinol Metab (2006) 91(7):2587–91. doi: 10.1210/jc.2005-1603 16621910

[10] Dhillon-Smith RK Middleton LJ Sunner KK Cheed V Baker K Farrell-Carver S. Levothyroxine in women with thyroid peroxidase antibodies before conception. New Engl J Med (2019) 380(14):1316–25. doi: 10.1056/NEJMoa1812537 30907987

[11] Zhu Q Xu QH Xie T Wang LL Liu H Muyayalo KP. Recent insights into the impact of immune dysfunction on reproduction in autoimmune thyroiditis. Clin Immunol (2021) 224:108663. doi: 10.1016/j.clim.2020.108663 33401032

[12] Weetman AP. Immunity, thyroid function and pregnancy: molecular mechanisms. Nat Rev Endocrinol (2010) 6(6):311–8. doi: 10.1038/nrendo.2010.46 20421883

[13] Monteleone P Parrini D Faviana P Carletti E Casarosa E Uccelli A. Female infertility related to thyroid autoimmunity: The ovarian follicle hypothesis. Am J Reprod Immunol (2011) 66(2):108–14. doi: 10.1111/j.1600-0897.2010.00961.x 21241400

[14] Kelkar RL Meherji PK Kadam SS Gupta SK Nandedkar TD. Circulating auto-antibodies against the zona pellucida and thyroid microsomal antigen in women with premature ovarian failure. J Reprod Immunol (2005) 66(1):53–67. doi: 10.1016/j.jri.2005.02.003 15949562

[15] Matalon ST Blank M Levy Y Carp HJA Arad A Burek L. The pathogenic role of anti-thyroglobulin antibody on pregnancy: Evidence from an active immunization model in mice. Hum Reproduction (2003) 18(5):1094–9. doi: 10.1093/humrep/deg210 12721190

[16] Negro R Stagnaro-Green A. Diagnosis and management of subclinical hypothyroidism in pregnancy. BMJ (Clinical Res ed) (2014) 349:g4929. doi: 10.1136/bmj.g4929 25288580

[17] Sheehan PM Nankervis A Araujo Júnior E Da Silva Costa F. Maternal thyroid disease and preterm birth: Systematic review and meta-analysis. J Clin Endocrinol Metab (2015) 100(11):4325–31. doi: 10.1210/jc.2015-3074 26383905

[18] Maraka S Ospina NM O'Keeffe DT Espinosa De Ycaza AE Gionfriddo MR Erwin PJ. Subclinical hypothyroidism in pregnancy: A systematic review and meta-analysis. Thyroid. (2016) 26(4):580–90. doi: 10.1089/thy.2015.0418 PMC4827301 26837268

[19] Korevaar TI de Rijke YB Chaker L Medici M Jaddoe VW Steegers EA. Stimulation of thyroid function by human chorionic gonadotropin during pregnancy: A risk factor for thyroid disease and a mechanism for known risk factors. Thyroid. (2017) 27(3):440–50. doi: 10.1089/thy.2016.0527 28049387

[20] Korevaar TI Steegers EA Pop VJ Broeren MA Chaker L de Rijke YB. Thyroid autoimmunity impairs the thyroidal response to human chorionic gonadotropin: Two population-based prospective cohort studies. J Clin Endocrinol Metab (2017) 102(1):69–77. doi: 10.1210/jc.2016-2942. 27754809

[21] Loh TP Tee JC Tee NW Cheng WL Thevarajah M Sabir N. Association between thyroid function tests and anti-thyroid peroxidase (TPO) antibodies in pregnancy. Endocrine. (2016) 53(3):865–7. doi: 10.1007/s12020-015-0844-y 26725315

[22] Chan S Boelaert K. Optimal management of hypothyroidism, hypothyroxinaemia and euthyroid TPO antibody positivity preconception and in pregnancy. Clin Endocrinol (Oxf) (2015) 82(3):313–26. doi: 10.1111/cen.12605 25200555

[23] Korevaar TIM Medici M Visser TJ Peeters RP. Thyroid disease in pregnancy: new insights in diagnosis and clinical management. Nat Rev Endocrinol (2017) 13(10):610–22. doi: 10.1038/nrendo.2017.93 28776582

[24] Kim NY Cho HJ Kim HY Yang KM Ahn HK Thornton S. Thyroid autoimmunity and its association with cellular and humoral immunity in women with reproductive failures. Am J Reprod Immunol (2011) 65(1):78–87. doi: 10.1111/j.1600-0897.2010.00911.x 20712806

[25] Cellini M Santaguida MG Stramazzo I Capriello S Brusca N Antonelli A. Recurrent pregnancy loss in women with hashimoto's thyroiditis with concurrent non-endocrine autoimmune disorders. Thyroid. (2020) 30(3):457–62. doi: 10.1089/thy.2019.0456 31910128

[26] Caccavo D Pellegrino NM Nardelli C Vergine S Leone L Marolla A. Anti-laminin-1 antibodies in serum and follicular fluid of women with hashimoto's thyroiditis undergoing *in vitro* fertilization. Int J Immunopathol Pharmacol (2016) 29(2):280–7. doi: 10.1177/0394632015627281 PMC5806718 26813862

[27] Volpé R. B-lymphocytes in autoimmune thyroid diseases (AITD). Rev Endocr Metab Disord (2000) 1(1-2):79–86. doi: 10.1023/A:1010068504848 11704995

[28] Weetman AP. Autoimmune thyroiditis: predisposition and pathogenesis. Clin Endocrinol (Oxf) (1992) 36(4):307–23. doi: 10.1111/j.1365-2265.1992.tb01453.x 1424162

[29] Rawlings DJ Metzler G Wray-Dutra M Jackson SW. Altered b cell signalling in autoimmunity. Nat Rev Immunol (2017) 17(7):421–36. doi: 10.1038/nri.2017.24 PMC5523822 28393923

[30] Arbuckle MR McClain MT Rubertone MV Scofield RH Dennis GJ James JA. Development of autoantibodies before the clinical onset of systemic lupus erythematosus. New Engl J Med (2003) 349(16):1526–33. doi: 10.1056/NEJMoa021933 14561795

[31] Deane KD Norris JM Holers VM. Preclinical rheumatoid arthritis: identification, evaluation, and future directions for investigation. Rheum Dis Clin North Am (2010) 36(2):213–41. doi: 10.1016/j.rdc.2010.02.001 PMC2879710 20510231

[32] Sokolove J Bromberg R Deane KD Lahey LJ Derber LA Chandra PE. Autoantibody epitope spreading in the pre-clinical phase predicts progression to rheumatoid arthritis. PLoS One (2012) 7(5):e35296. doi: 10.1371/journal.pone.0035296 22662108 PMC3360701

[33] Pihoker C Gilliam LK Hampe CS Lernmark A. Autoantibodies in diabetes. Diabetes. (2005) 54 Suppl 2:S52–61. doi: 10.2337/diabetes.54.suppl_2.S52 16306341

[34] Packard TA Cambier JC. B lymphocyte antigen receptor signaling: initiation, amplification, and regulation. F1000Prime Rep (2013) 5:40. doi: 10.12703/P5-40 24167721 PMC3790562

[35] Johnson SA Pleiman CM Pao L Schneringer J Hippen K Cambier JC. Phosphorylated immunoreceptor signaling motifs (ITAMs) exhibit unique abilities to bind and activate Lyn and syk tyrosine kinases. J Immunol (Baltimore Md 1950) (1995) 155(10):4596–603. 7594458

[36] Saijo K Schmedt C Su IH Karasuyama H Lowell CA Reth M. Essential role of src-family protein tyrosine kinases in NF-kappaB activation during b cell development. Nat Immunol (2003) 4(3):274–9. doi: 10.1038/ni893 12563261

[37] Gold MR Matsuuchi L Kelly RB DeFranco AL. Tyrosine phosphorylation of components of the b-cell antigen receptors following receptor crosslinking. Proc Natl Acad Sci United States America (1991) 88(8):3436–40. doi: 10.1073/pnas.88.8.3436 PMC51462 1707541

[38] Rowley RB Burkhardt AL Chao HG Matsueda GR Bolen JB. Syk protein-tyrosine kinase is regulated by tyrosine-phosphorylated Ig alpha/Ig beta immunoreceptor tyrosine activation motif binding and autophosphorylation. J Biol Chem (1995) 270(19):11590–4. doi: 10.1074/jbc.270.19.11590 7538118

[39] Coggeshall KM Cambier JC. B cell activation. VIII. membrane immunoglobulins transduce signals *via* activation of phosphatidylinositol hydrolysis. J Immunol (Baltimore Md 1950) (1984) 133(6):3382–6. 6092473

[40] Ransom JT Harris LK Cambier JC. Anti-ig induces release of inositol 1,4,5-trisphosphate, which mediates mobilization of intracellular ca++ stores in b lymphocytes. J Immunol (Baltimore Md 1950) (1986) 137(2):708–14. 3014002

[41] Hogan PG Lewis RS Rao A. Molecular basis of calcium signaling in lymphocytes: STIM and ORAI. Annu Rev Immunol (2010) 28:491–533. doi: 10.1146/annurev.immunol.021908.132550 20307213 PMC2861828

[42] Penna A Demuro A Yeromin AV Zhang SL Safrina O Parker I. The CRAC channel consists of a tetramer formed by stim-induced dimerization of orai dimers. Nature. (2008) 456(7218):116–20. doi: 10.1038/nature07338 PMC2597643 18820677

[43] Berry CT Liu X Myles A Nandi S Chen YH Hershberg U. BCR-induced Ca2+ signals dynamically tune survival, metabolic reprogramming, and proliferation of naive b cells. Cell Rep (2020) 31(2):107474. doi: 10.1016/j.celrep.2020.03.038 32294437 PMC7301411

[44] Baba Y Kurosaki T. Role of Calcium Signaling in B Cell Activation and Biology. Curr Top Microbiol Immunol (2016) 393:143–74. doi: 10.1007/82_2015_477 26369772

[45] Jackson SW Kolhatkar NS Rawlings DJ. B cells take the front seat: dysregulated b cell signals orchestrate loss of tolerance and autoantibody production. Curr Opin Immunol (2015) 33:70–7. doi: 10.1016/j.coi.2015.01.018 PMC4397155 25679954

[46] Hwangbo Y Park YJ. Genome-wide association studies of autoimmune thyroid diseases, thyroid function, and thyroid cancer. Endocrinol Metab (Seoul) (2018) 33(2):175–84. doi: 10.3803/EnM.2018.33.2.175 PMC6021314 29947174

[47] Hom G Graham RR Modrek B Taylor KE Ortmann W Garnier S. Association of systemic lupus erythematosus with C8orf13-BLK and ITGAM-ITGAX. New Engl J Med (2008) 358(9):900–9. doi: 10.1056/NEJMoa0707865 18204098

[48] Lu R Vidal GS Kelly JA Delgado-Vega AM Howard XK Macwana SR. Genetic associations of LYN with systemic lupus erythematosus. Genes Immun (2009) 10(5):397–403. doi: 10.1038/gene.2009.19 19369946 PMC2750001

[49] Harley JB Alarcón-Riquelme ME Criswell LA Jacob CO Kimberly RP Moser KL. Genome-wide association scan in women with systemic lupus erythematosus identifies susceptibility variants in ITGAM, PXK, KIAA1542 and other loci. Nat Genet (2008) 40(2):204–10. doi: 10.1038/ng.81 PMC3712260 18204446

[50] Armitage LH Wallet MA Mathews CE. Influence of PTPN22 allotypes on innate and adaptive immune function in health and disease. Front Immunol (2021) 12(189). doi: 10.3389/fimmu.2021.636618 PMC7946861 33717184

[51] Wu H Wan S Qu M Ren B Liu L Shen H. The relationship between PTPN22 R620W polymorphisms and the susceptibility to autoimmune thyroid diseases: An updated meta-analysis. Immunol Investigations (2020) 51 (2): 438–451. doi: 10.1080/08820139.2020.1837154 33103521

[52] Xiaoheng C Yizhou M Bei H Huilong L Xin W Rui H. General and specific genetic polymorphism of cytokines-related gene in AITD. Mediators inflamm (2017) 2017:3916395–. doi: 10.1155/2017/3916395 PMC5241475 28133421

[53] Arechiga AF Habib T He Y Zhang X Zhang Z-Y Funk A. Cutting edge: the PTPN22 allelic variant associated with autoimmunity impairs b cell signaling. J Immunol (Baltimore Md 1950) (2009) 182(6):3343–7. doi: 10.4049/jimmunol.0713370 PMC2797545 19265110

[54] Dai X James RG Habib T Singh S Jackson S Khim S. A disease-associated PTPN22 variant promotes systemic autoimmunity in murine models. J Clin Invest (2013) 123(5):2024–36. doi: 10.1172/JCI66963 PMC3638909 23619366

[55] Wu YC Kipling D Dunn-Walters DK. The relationship between CD27 negative and positive b cell populations in human peripheral blood. Front Immunol (2011) 2:81. doi: 10.3389/fimmu.2011.00081 22566870 PMC3341955

[56] Nevalainen T Autio A Kummola L Salomaa T Junttila I Jylhä M. CD27- IgD- b cell memory subset associates with inflammation and frailty in elderly individuals but only in males. Immun Ageing (2019) 16:19. doi: 10.1186/s12979-019-0159-6 31423147 PMC6693136

[57] Wei C Anolik J Cappione A Zheng B Pugh-Bernard A Brooks J. A new population of cells lacking expression of CD27 represents a notable component of the b cell memory compartment in systemic lupus erythematosus. J Immunol (Baltimore Md 1950) (2007) 178(10):6624–33. doi: 10.4049/jimmunol.178.10.6624 17475894

[58] Brisslert M Bokarewa M Larsson P Wing K Collins LV Tarkowski A. Phenotypic and functional characterization of human CD25+ b cells. Immunology. (2006) 117(4):548–57. doi: 10.1111/j.1365-2567.2006.02331.x PMC1782245 16556269

[59] Habib T Funk A Rieck M Brahmandam A Dai X Panigrahi AK. Altered b cell homeostasis is associated with type I diabetes and carriers of the PTPN22 allelic variant. J Immunol (Baltimore Md 1950) (2012) 188(1):487–96. doi: 10.4049/jimmunol.1102176 PMC3670766 22105996

[60] Kolhatkar NS Brahmandam A Thouvenel CD Becker-Herman S Jacobs HM Schwartz MA. Altered BCR and TLR signals promote enhanced positive selection of autoreactive transitional b cells in wiskott-Aldrich syndrome. J Exp Med (2015) 212(10):1663–77. doi: 10.1084/jem.20150585 PMC4577851 26371186

[61] Thien M Phan TG Gardam S Amesbury M Basten A Mackay F. Excess BAFF rescues self-reactive b cells from peripheral deletion and allows them to enter forbidden follicular and marginal zone niches. Immunity. (2004) 20(6):785–98. doi: 10.1016/j.immuni.2004.05.010 15189742

[62] Lesley R Kelly LM Xu Y Cyster JG. Naive CD4 T cells constitutively express CD40L and augment autoreactive b cell survival. Proc Natl Acad Sci United States America (2006) 103(28):10717–22. doi: 10.1073/pnas.0601539103 PMC1484418 16815973

[63] Lesley R Xu Y Kalled SL Hess DM Schwab SR Shu HB. Reduced competitiveness of autoantigen-engaged b cells due to increased dependence on BAFF. Immunity. (2004) 20(4):441–53. doi: 10.1016/S1074-7613(04)00079-2 15084273

[64] Giltiay NV Giordano D Clark EA. The plasticity of newly formed b cells. J Immunol (2019) 203(12):3095–104. doi: 10.4049/jimmunol.1900928 PMC6961804 31818922

[65] Meffre E Wardemann H. B-cell tolerance checkpoints in health and autoimmunity. Curr Opin Immunol (2008) 20(6):632–8. doi: 10.1016/j.coi.2008.09.001 18848883

[66] William J Euler C Christensen S Shlomchik MJ. Evolution of autoantibody responses *via* somatic hypermutation outside of germinal centers. Sci (New York NY) (2002) 297(5589):2066–70. doi: 10.1126/science.1073924 12242446

