# Supplementary material for: B cells from anti-thyroid antibody positive, infertile women show hyper-reactivity to BCR stimulation
Source: Front Immunol. 2022 Oct 25;13:1039166. doi: 10.3389/fimmu.2022.1039166 (PMC9641243; doi:10.3389/fimmu.2022.1039166)
Supplement: Supplementary file 1 [file DataSheet_1.pdf]

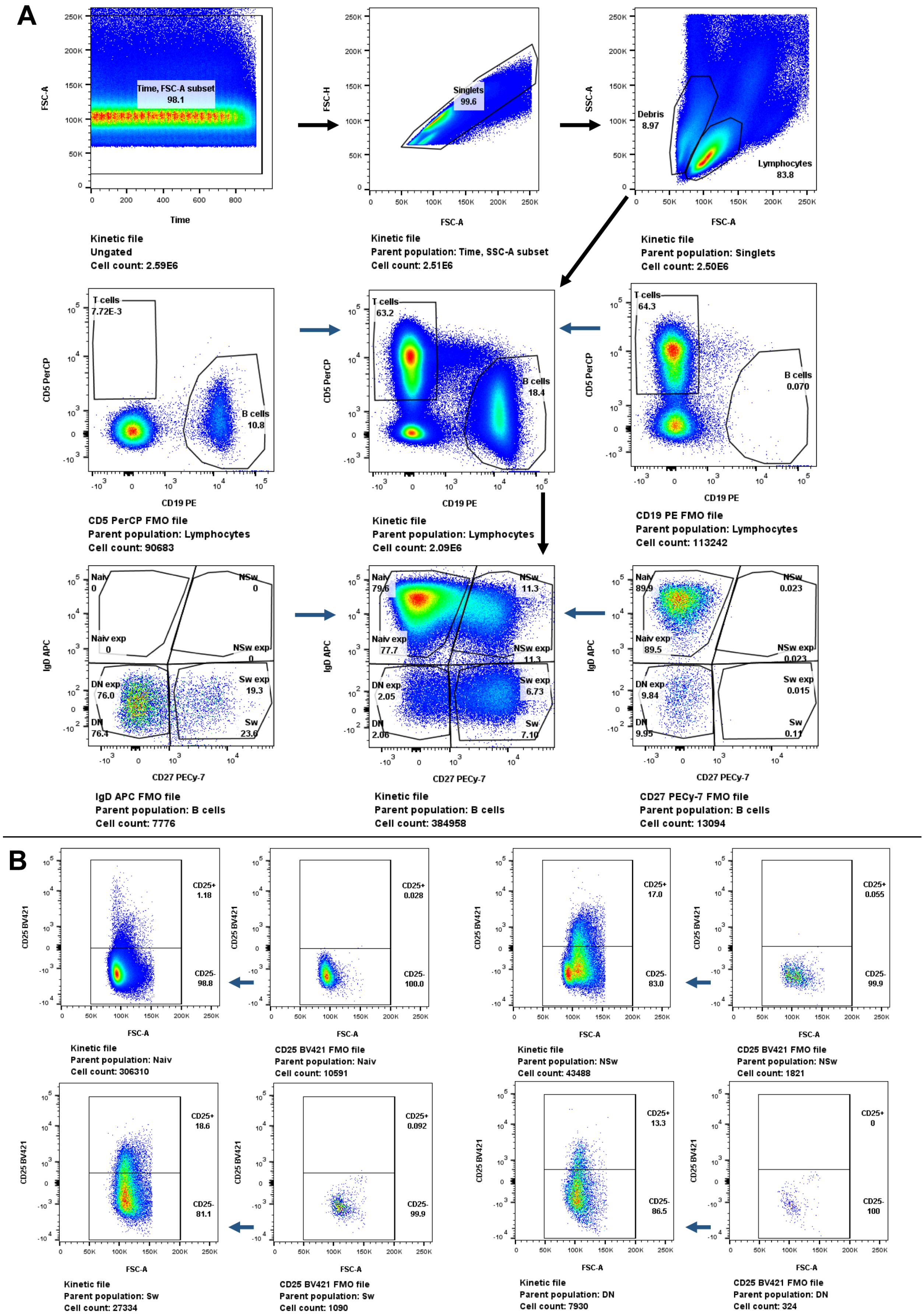

Supplementary Figure 1. The gating strategy used to identify the main B cell subsets

Arrows are used to visualize the relationships across plots. The gates are always set based on FMO (fluorescent minus one) controls.

A. Time versus FSC-A gate was set to exclude fluorescence intensity fluctuations of the flow cytometer. Gate for doublet discrimination was used based on FSC-H/FSC-A. Single cells were further defined based on SSC-A and FSC-A. The lymphocyte population is then selected along the FSC and SSC parameters. Within the lymphocyte population, CD3+ CD19- cells represent the T cell population and the CD3- CD19 + population represents B lymphocytes. Expression of CD27 and IgD was used to classify: naive B cells (IgD+CD27−), non-switched memory B cells (IgD+CD27+; NSw), switched memory B cells (IgD−CD27+; Sw), and IgD and CD27 double-negative B cells (IgD−CD27−; DN).

B. Within each subgroup, we examined the level of CD25 expression.

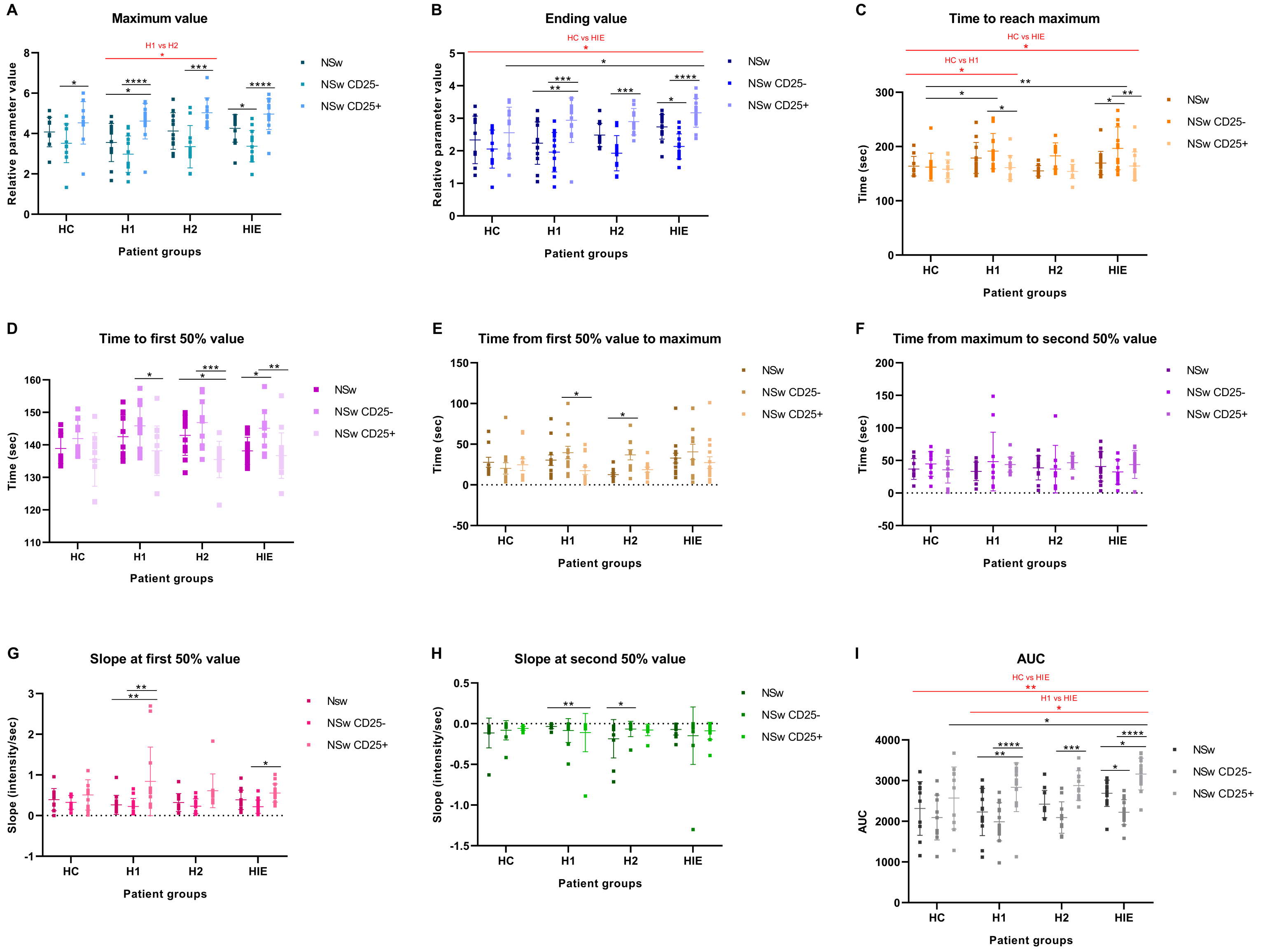

Supplementary Figure 2. Kinetic parameters of the calcium flux response of non-switched memory B cells

Calcium flux kinetic data of non-switched (NSw) memory B cells, CD25- and CD25+ NSw cells (A) maximum value, (B) ending value, (C) time to reach maximum, (D) time to first 50% value, (E) time from first 50% to maximum, (F) time from maximum to second 50%, (G) slope at the first 50% value, (H) slope at second 50% value, (I) area under curve (AUC) value

HC: healthy controls (n=12); HT: Hashimoto's thyroiditis, H1: hypothyroid patients with HT before treatment (n=14); H2: euthyroid patients with HT, after levothyroxine-treatment, (n=12); HIE: HT, infertile, euthyroid patients (n=14).

The kinetic data were analyzed by the FACSKin software. Differences between the groups were assessed with two-way ANOVA and Tukey's post hoc test except H1-H2 comparisons where a mixed-effects analysis and the Tukey's multiple comparisons test were used because these groups represent the same individuals in a repeated sampling before and during treatment. Data are depicted as individual values, middle line represents the mean, whiskers are set to standard deviation (SD) *p<0.05, **p<0.01, ***p<0.001, ****p<0.0001.

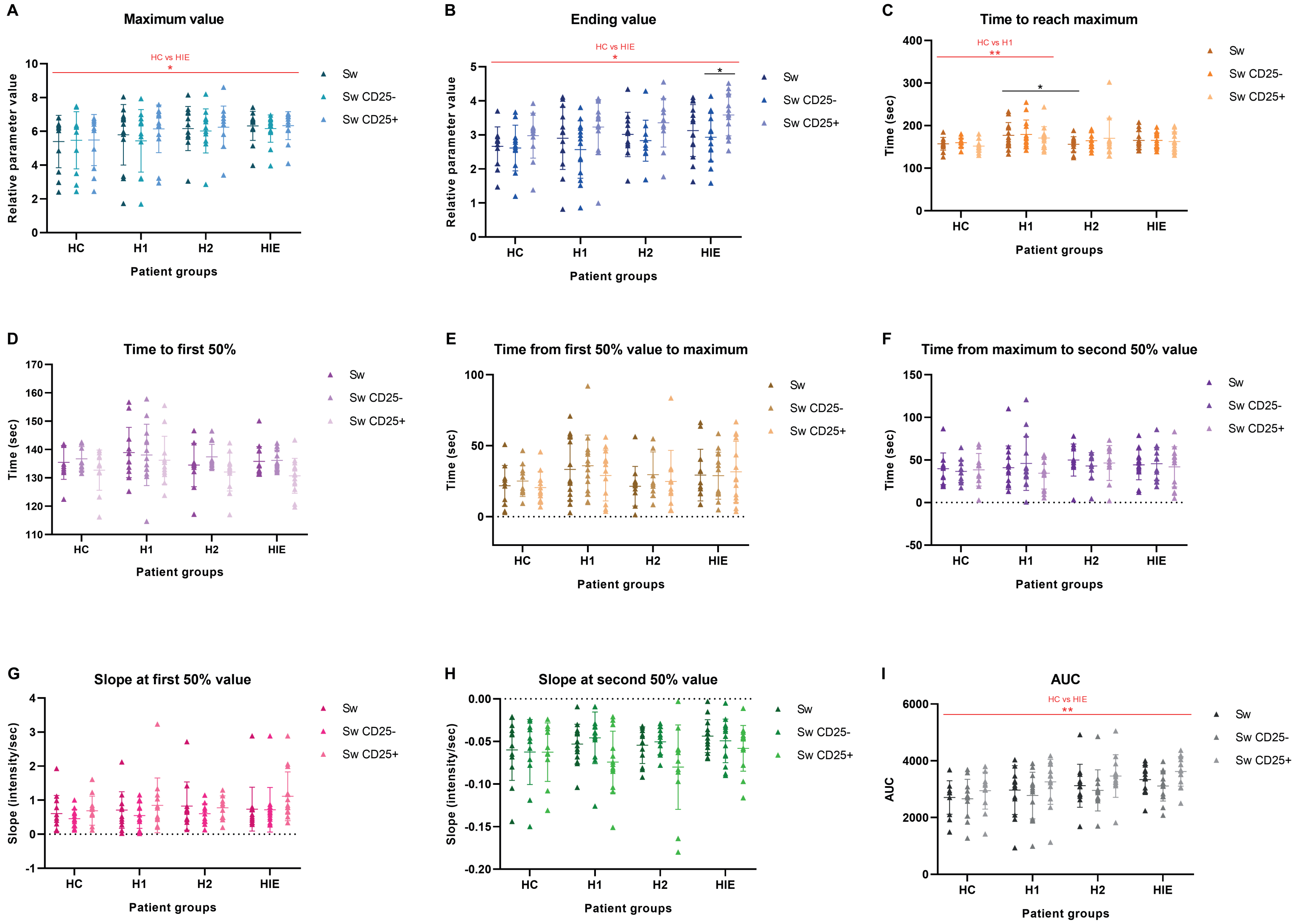

Supplementary Figure 3. Kinetic parameters of the calcium flux response of switched memory B cells

Calcium flux kinetic data of immunoglobulin class-switched (Sw) memory B cells, CD25- and CD25+ Sw cells (A) maximum value, (B) ending value, (C) time to reach maximum, (D) time to first 50% value, (E) time from first 50% to maximum, (F) time from maximum to second 50%, (G) slope at the first 50% value, (H) slope at second 50% value, (I) area under curve (AUC) value

HC: healthy controls (n=12); HT: Hashimoto's thyroiditis, H1: hypothyroid patients with HT before treatment (n=14); H2: euthyroid patients with HT, after levothyroxine-treatment, (n=12); HIE: HT, infertile, euthyroid patients (n=14).

The kinetic data were analyzed by the FACSKin software. Differences between the groups were assessed with two-way ANOVA and Tukey's post hoc test except H1-H2 comparisons where a mixed-effects analysis and the Tukey's multiple comparisons test were used because these groups represent the same individuals in a repeated sampling before and during treatment. Data are depicted as individual values, middle line represents the mean, whiskers are set to standard deviation (SD). *$p<0.05$, **$p<0.01$, ***$p<0.001$, ****$p<0.0001$.

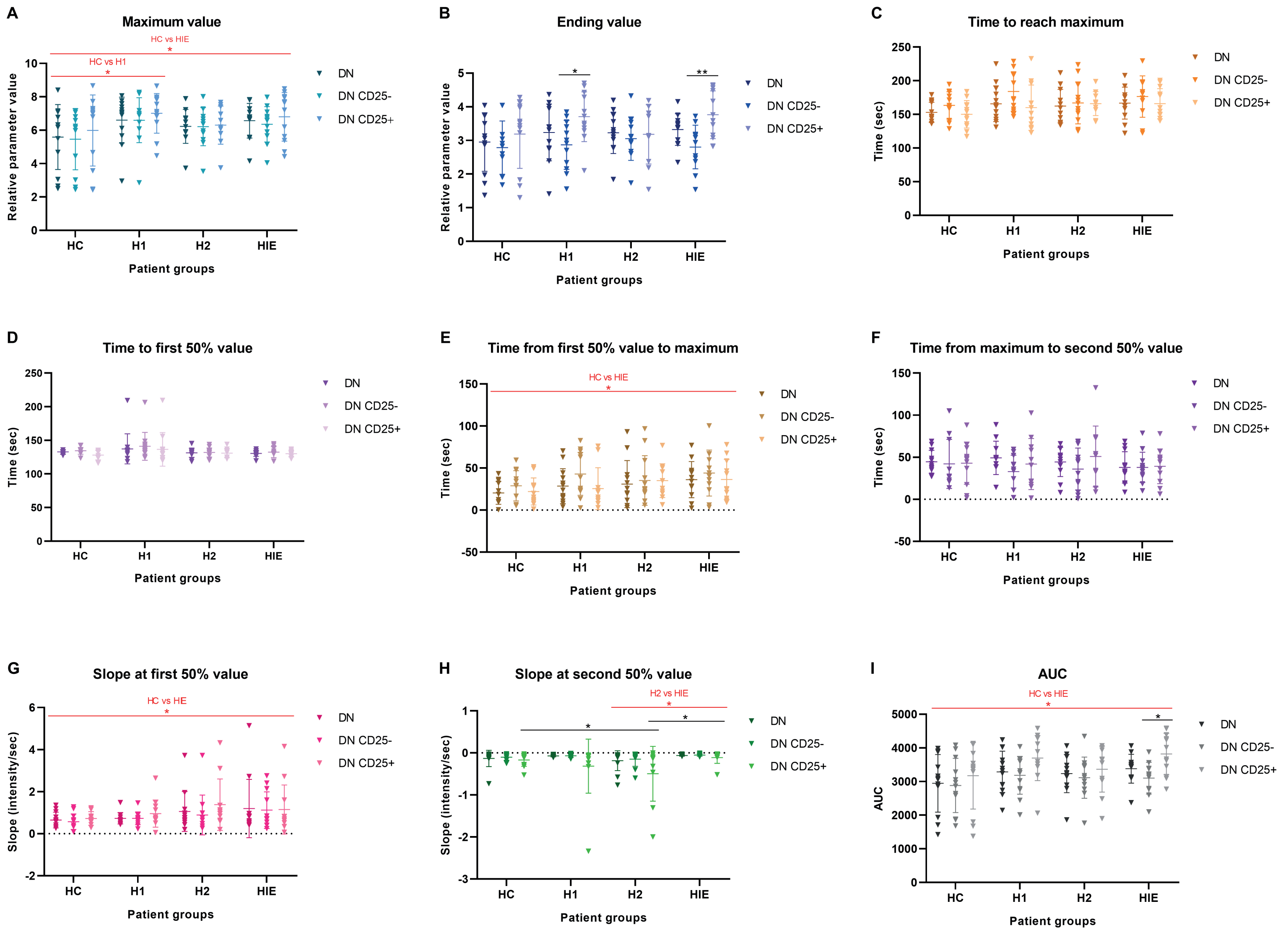

Supplementary Figure 4. Kinetic parameters of the calcium flux response of double negative memory B cells

Calcium flux kinetic data of CD27-, IgD- double-negative (DN) memory B cells, CD25- and CD25+ DN cells (A) maximum value, (B) ending value, (C) time to reach maximum, (D) time to first 50% value, (E) time from first 50% to maximum, (F) time from maximum to second 50%, (G) slope at the first 50% value, (H) slope at second 50% value, (I) area under curve (AUC) value

HC: healthy controls (n=12); HT: Hashimoto's thyroiditis, H1: hypothyroid patients with HT before treatment (n=14); H2: euthyroid patients with HT, after levothyroxine-treatment, (n=12); HIE: HT, infertile, euthyroid patients (n=14).

The kinetic data were analyzed by the FACSKin software. Differences between the groups were assessed with two-way ANOVA and Tukey's post hoc test except H1-H2 comparisons where a mixed-effects analysis and the Tukey's multiple comparisons test were used because these groups represent the same individuals in a repeated sampling before and during treatment. Data are depicted as individual values, middle line represents the mean, whiskers are set to standard deviation (SD). $*p<0.05$, $**p<0.01$, $***p<0.001$, $****p<0.0001$.
